# Shared Decision Making With Young People at Ultra High Risk of Psychotic Disorder

**DOI:** 10.3389/fpsyt.2021.683775

**Published:** 2021-09-16

**Authors:** Magenta Bender Simmons, Mary Brushe, Aurora Elmes, Andrea Polari, Barnaby Nelson, Alice Montague

**Affiliations:** ^1^Orygen, Parkville, VIC, Australia; ^2^Centre for Youth Mental Health, The University of Melbourne, Parkville, VIC, Australia; ^3^Telethon Kids Institute, University of Western Australia, Adelaide, SA, Australia; ^4^School of Public Health, University of Adelaide, Adelaide, SA, Australia; ^5^Centre for Social Impact, Swinburne University of Technology, Hawthorn, VIC, Australia; ^6^North East London Foundation NHS Trust, London, United Kingdom; ^7^Research Department of Clinical, Educational and Health Psychology, University College London, London, United Kingdom

**Keywords:** shared decision making, ultra high risk, clinical high risk, at risk mental states, youth, early intervention, psychosis

## Abstract

**Introduction:** While the majority of young people who meet the criteria for being considered at increased risk of psychosis do not go on to develop a psychotic disorder, young people are currently being identified and treated in early intervention services. Ethical concerns have been raised concerning the decision about whether or not to provide treatment, and if so, what type of treatment. This study sought to support young people themselves to make these decisions with support from their clinician through a shared decision-making approach, facilitated by an online decision aid.

**Methods:** This project used the International Patient Decision Aid Standards (IPDAS) to guide the development and piloting of an online decision aid across two phases: (1) qualitative, semi-structured focus groups with young people who were past clients and clinicians from an early psychosis service; and (2) pilot testing of the decision aid with clinicians and young people who were current clients to finalize the development.

**Results:** Issues discussed by clinicians in the focus group were grouped into three main areas: (1) engagement phase; (2) assessment and priorities for treatment; and (3) initial and ongoing decision making. Clients focused on the context in which the decisions were made, including as they experienced initial feelings of resistance, and then acceptance of efforts made to describe and treat their mental health challenges. Clients highlighted the need for collaboration between themselves and their clinician, and the need to be equipped with the knowledge and tools to take care of themselves. These focus group data were used to refine the online decision aid. Pilot testing revealed that while it was overall useful and relevant, important limitations were noted by both clients and clinicians.

**Discussion:** The use of a decision aid to facilitate shared decision making (SDM) in this area is feasible and has utility for both clients and clinicians. Use of such a tool can help to address the need to uphold the rights of young people as decision makers about their own care. Future efforts should embed decision aids within complex SDM interventions, and research to understand issues relating to implementation of these interventions.

## Introduction

Approximately three in every 1,000 Australians will experience a first episode of psychosis (FEP) in any given year (1). Despite this relative low incidence, psychotic disorders can be highly debilitating, with a recent systematic review and meta-analysis suggesting those with a diagnosis of schizophrenia experience an average of 14.5 years of potential life lost (2). Psychotic disorders are also associated with an increased risk of homelessness (3), decreased social functioning (4), and higher rates of suicide (5) and unemployment (6, 7). Given the potential impact of psychosis, efforts have been made to identify individuals most at risk in order to intervene early in the hope of delaying, ameliorating, or even preventing the onset of a disorder (8).

The main way in which risk has been operationalized is through the development of the Ultra High Risk (UHR) criteria (9). To meet the UHR criteria, a young person must have experienced either (1) a 30% or greater drop in functioning sustained for 1 month within the past 12 months, or (2) chronically low functioning for the past 12 months or longer, and also fall into one of the following risk groups: (1) Vulnerability Group (those with either a family history of psychosis or a diagnosis of Schizotypal Personality Disorder); (2) Attenuated Psychosis Group [sub-threshold positive psychotic symptoms in the past year as assessed using the Comprehensive Assessment of At-Risk Mental States (10)]; and (3) Brief Limited Intermittent Psychosis Syndrome (BLIPS) Group (experienced episodes of psychotic symptoms within the past year that have not lasted longer than a week and have resolved without treatment).

For young people meeting the UHR criteria, the cumulative risk of transitioning to a psychotic disorder is estimated to be approximately 19% at 2 year follow-up, and 36.5% at 10–11 year follow-up (11). Other longitudinal research indicates that individuals who met UHR criteria but did not go on to develop psychosis still experienced significant, persistent negative symptoms, mood and anxiety concerns, low rates of employment, and high suicide rates in comparison to those with first episode psychosis and their peers who do not meet UHR criteria (12). Together, these findings highlight that young people who meet the UHR criteria require early intervention for mental health challenges, regardless of whether they experience a psychotic episode.

Randomized controlled trials have investigated cognitive behavior therapy (CBT), anti-psychotic medications, and Omega-3 fatty acids as interventions for those who meet UHR criteria aiming to reduce the risk of transition to psychosis (13). Recent systematic reviews and meta-analyses have demonstrated CBT as the most effective intervention in comparison to controls at reducing transition to psychosis, however, a network meta-analysis comparing multiple interventions (CBT, integrated psychological therapy, omega-3, family therapy, ziprasidone, needs-based interventions, risperidone plus CBT, and olanzapine), showed no intervention was more effective than another (14–16). However, systematic review evidence indicates that interventions in this population are cost-effective, and can lead to cost-savings (17), however the results were limited by the heterogeneity of services and a lack of consensus on the best practice for intervention in the UHR population.

Current Australian clinical guidelines (18) recommend CBT as the preferred intervention for young people meeting UHR criteria, but note antipsychotic medications may be provided if a person is considered to have experienced a psychotic episode (i.e., more than 1 week of frank positive psychotic symptoms have been sustained) or if milder positive symptoms associated with risk of self-harm or aggression are present. Psychoeducation about psychosis, their risk of developing psychosis, and what evidence-based treatment options are available, with consideration of client preference, is also recommended (19). The guidelines also indicate Omega-3 fatty acids may delay or prevent transition to psychosis, however the finding underpinning this recommendation has not been replicated in a larger trial (20). It should be noted, there has been considerable debate in the field over the last decade on the ethics of labeling and intervening on young people at UHR (21–25). A major focus of the debate is on whether treatment should involve a focus on the clients presenting problems or their psychosis-risk symptoms (24, 25). One approach which has been argued for as imperative to ensuring that treatment decisions are evidence-based and preference-sensitive for young people at UHR is shared decision making (SDM) (19).

Shared decision making is a collaborative approach to treatment decision making with roots in both evidence-based medicine and client-centered care (26). Decision aids are the most common way to facilitate SDM; decision aids describe the different treatment options relevant to the decision and present evidence-based information about the potential harms and benefits of each option, and the likelihood of these outcomes. They also elicit personal preferences and values so that the person faced with the decision can work together with their treating clinician or team and any caregivers involved in their care. Decision aids have demonstrated effectiveness in increasing client knowledge, reducing decisional conflict (both in terms of feeling uninformed or feeling unclear about personal values), reducing the proportion of clients who are passive in the decision-making process, and reducing the proportion of clients who remain undecided about what treatment option to choose (27).

Systematic reviews within the mental health field have led to recommendations for decision aids, along with other approaches to facilitate SDM, to be used within mental health treatment settings, although most of the research to date has focused on adult populations (28–30). A systematic review of SDM specifically with psychiatric patients found that SDM interventions were associated with a small overall increase in indices of empowerment such as patients' subjective sense of involvement in treatment, self-efficacy, and autonomy (31), with a trend toward reducing the likelihood of compulsory inpatient treatment over 15–18 months. However, authors acknowledge the data were heterogeneous and imprecise, highlighting a need for high quality studies in this area (31). A more recent clinical review found SDM to be particularly important when considering drug treatments for patients with schizophrenia, although research to date lacks data on the stability and maintenance of positive effects over time (32). Despite the increasing focus in this area, there is still a paucity of research focused on youth populations.

Incorporating SDM in youth mental health settings, with young people aged 15–24 years, may be beneficial in managing complexities arising from agreeing treatment plans between young people, caregivers, and clinicians, especially considering the ethical and legal issues associated with treatment consent (33). Service providers are already beginning to introduce SDM practices with young people (34) however, the effectiveness of these approaches have limited empirical evidence. One study found that an intervention that combined peer work led to an increased sense of involvement in their assessment and lower decisional conflict, both of which are important components of client satisfaction (35). Another study in young people with depression trialed a collaborative care intervention which included aspects of SDM, and demonstrated greater improvements in depressive symptoms 12 months later (36). One study developed an Encounter Decision Aid and piloted it with patients with first-episode psychosis and long-term psychosis, family members, and clinicians (37). The decision aid was found to be valuable and acceptable, however the research did not exclusively focus on young people and excluded any patients under the age of 18. Of interest, a recent protocol has been published that aims to evaluate the feasibility of a decision aid to promote SDM among young adults with first-episode psychosis, but results are yet to be published (38). To date, there have been no studies specifically focused on SDM approaches with young people, inclusive of those under the age of 18, who are accessing UHR services, despite the clinical guidelines recommending clinicians utilize SDM (18).

The current study focuses on empowering young people meeting UHR criteria to become active participants in their own care by involving them in the treatment decision-making process. We describe the development of an online decision aid that presents the evidence for treatment options according to the Australian Clinical Guidelines for Early Psychosis and is designed to be used with young people and their treating clinician. This study reports on the development and piloting of this decision aid. We sought to answer the following research question: do clients and clinicians find the decision aid relevant and useful, and does it result in clients feeling satisfied with the decision and have low decisional conflict?

## Methods

This study used the International Patient Decision Aid Standards (IPDAS) (39) to guide the development of the decision aid across two phases. Phase one involved using the IPDAS to generate a prototype decision aid, which was then used as a prompt in qualitative focus groups both with young people who had previously accessed early psychosis services and clinicians working at such a service. The results of these data then informed phase two, which involved refining the content and design of the online decision aid and piloting it with a small number of clinicians and clients to finalize the development process (example screen shot of the final version presented in Supplementary Material 1 and study timeline presented in Supplementary Material 2). This study was approved by the Melbourne Health Human Research Ethics Committee (reference 2014.155).

### Setting

This study was conducted at the Early Psychosis Prevention and Intervention Center (EPPIC) and the Personal Assessment and Crisis Evaluation (PACE) Clinic, both of which are part of Orygen Specialist Program (OSP). Orygen Specialist Program is a tertiary mental health service that provides mental health care to young people aged 15–24 in the north-western metropolitan area of Melbourne in the state of Victoria, Australia. It runs a range of clinics for young people with emerging and established mental disorders (including EPPIC and PACE) and provides both outpatient and inpatient care. Orygen Specialist Program has a consumer reference group called Platform; Platform members are young people who have been discharged from OSP and engage in activities to contribute to the ongoing improvement of the service, improve mental health literacy and help seeking in young people, and reduce stigma around mental illness (40, 41).

### Phase One: Qualitative Focus Groups With Stakeholders to Refine the Decision Aid

The development of the content and design of the initial version of the decision aid was conducted in accordance with the International Patient Decision Aids Standards (IPDAS) (39). The IPDAS are a set of theory-driven and empirically-informed standards that provide guidance on how to develop decision aids to maximize the chances of providing effective decision-making support and reduce the risk of introducing biases to the process. Two key decisions were identified to be supported by the decision aid, namely whether or not to seek help for meeting the UHR criteria and choice of intervention for those deciding to engage in treatment. The treatment options were based on the clinical practice guidelines (42), which at the time recommended omega-3 fatty acids (fish oil), cognitive behavioral therapy, supporting counseling, and support for mental health challenges in general. This early prototype was used as a prompt in two focus groups: the first with members from a consumer reference group (Platform), and the second with healthcare professionals working in the PACE Clinic. The focus groups lasted 67 and 55 min, respectively, and were co-facilitated by MS and AM using a semi-structured focus group schedule (see Supplementary Material 3 for example probes). Focus groups were audio recorded and transcribed verbatim. Data were analyzed using inductive thematic analysis (43), whereby thematic interpretations of the transcripts were derived directly from the text (44). Both AM and MS initially analyzed the data separately and then collaboratively, addressing any discrepancies through revisiting the data and discussion with the broader team, to support the validity of the analysis. Coding continued until no new themes were identified in the data (data saturation), and all responses could be explained in terms of the thematic structure.

### Phase Two: Pilot Testing

In order to complete the development process, pilot testing of the decision aid was conducted at the PACE Clinic with both clinicians and clients as participants. The clinician focus group in phase one was conducted with current clinicians, making it possible for clinician participants to participate in both phase one and two (which was the case for three clinicians), but not for clients, as phase one involved past clients and phase two involved current clients. Clinicians were recruited using convenience sampling through staff meetings and invited to nominate new clients of the service who were facing a decision about treatment options at the time. Clinicians were able to refer more than one client, and once a referral was made the research assistant contacted the client to provide more information and obtain informed consent if the client was willing to participate. Once this occurred, the decision aid was made available on a tablet device for use in the clinical appointment where treatment options were due to be discussed. After using the decision aid, both client and clinician were asked what decision was reached and why, and were invited to provide open-ended feedback about the usefulness, relevance, and appearance of each decision aid section and were asked for suggested changes.

Clients also completed the following measures to identify any extreme scores that could indicate the lack of utility of the decision aid:

1) Decisional conflict was measured with the Decisional Conflict Scale (DCS) (45). The DCS is a 16-item measure that uses a 0–4 Likert scale. It has a total score range of 0–100, where higher scores indicate higher decisional conflict (undesired outcome).
2) How satisfied participants were with the decision was measured with the Satisfaction With Decision (SWD) scale (46), a six-item 1–5 Likert scale self-report questionnaire with a maximum score of 30 where higher scores indicate higher satisfaction with the decision.

## Results

### Phase One: Qualitative Focus Groups With Stakeholders

In total, eight clinicians participated in the focus group, including allied health professionals (*n* = 6) and psychiatrists (*n* = 2). There were three males and five females, and age was not recorded. The Platform group (*n* = 6) included two young people who had been clients of PACE only (i.e., met the UHR criteria but not experienced a FEP); two young people who had been PACE clients and then transitioned to FEP and subsequently became EPPIC clients; and two young people who had been clients at EPPIC but who had not received treatment from PACE before experiencing FEP. There were two males and four females, with ages ranging from 18 to 29 years (mean = 25.5; SD = 3.94).

#### Clinicians' Experiences and Beliefs About Treatment Decision Making

**Figure 1:**
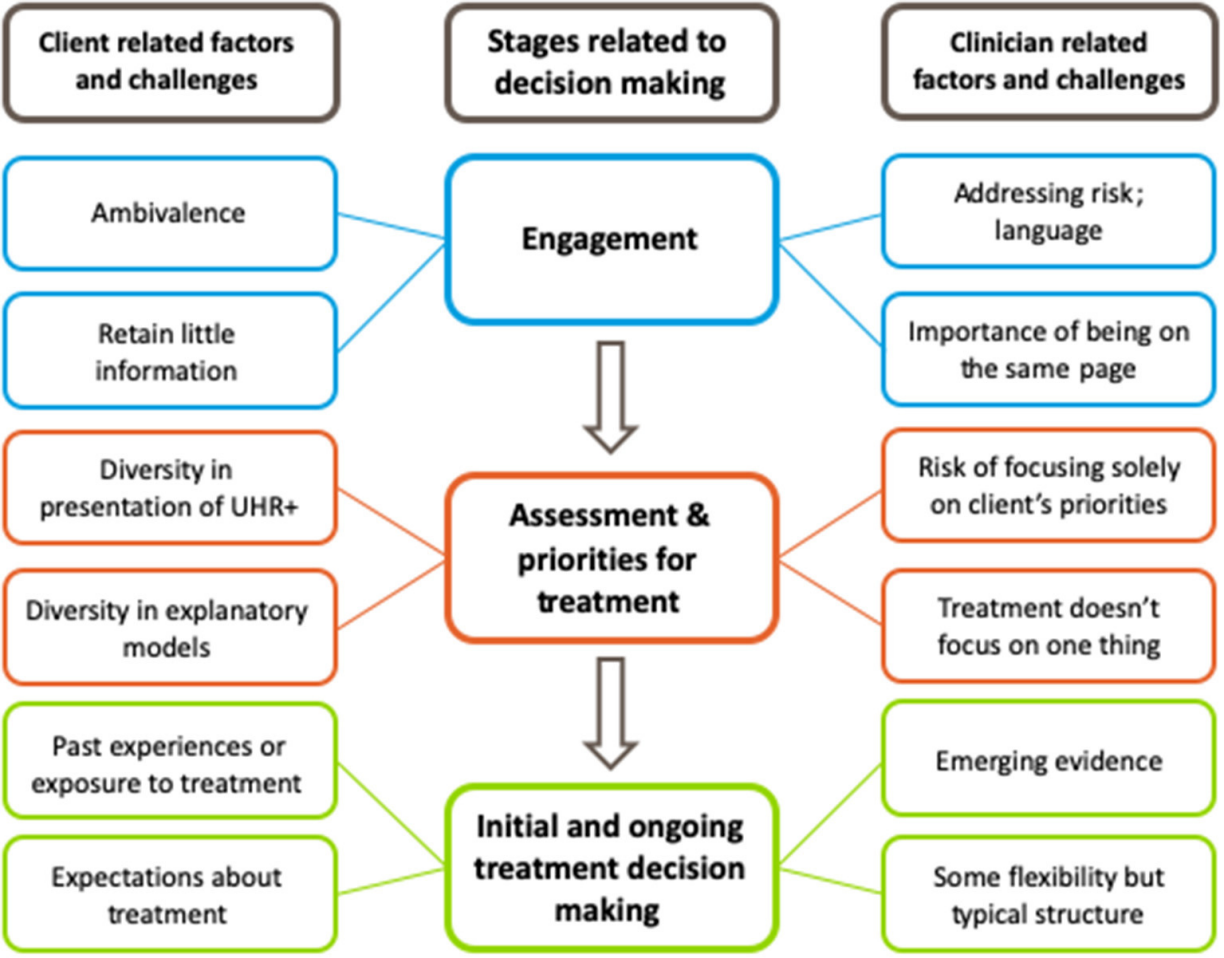
Thematic map of clinician reported experiences and beliefs about treatment decision making for young people who meet the “Ultra High Risk” (UHR) criteria.

When describing their experiences and beliefs about treatment decision making for young people meeting the UHR criteria, clinicians spoke about issues common to treatment decision making in youth mental health in general (e.g., tenuous clinical engagement). However, clinicians reported that these issues were heightened in this clinical population due to the lack of a formal diagnostic framework. Issues discussed by clinicians fell into three main areas: (1) engagement phase; (2) assessment and priorities for treatment; and (3) initial and ongoing decision making (see Figure 1).

#### Engagement Phase

Clinicians felt that deciding whether or not to engage in the service at all was the first decision faced by clients. Having information about the service was seen as a necessity at this stage, including what was “on offer” in terms of ways in which the client could be helped. The PACE Clinic was perceived differently to other youth mental health clinics, in that clients were referred with a “somewhat more subtle and ambiguous” rationale for treatment. This was seen as a factor that increased ambivalence of clients deciding whether or not to engage in treatment. Related to this was the concern that clients did not remember what was described about the service, psychoeducation about mental health, and information about treatment options.

“*Often, it's interesting, after you have your first one session, maybe two sessions, (and then you ask) ‘So why are you here? What do you know about PACE?' Despite having done a spiel and them seeming to engage, they very rarely retain any of that information.”*

Perceived reasons for this included clients facing “information overload”; having been through multiple services in their referral pathway to the PACE Clinic (“they have often bounced around a bit”); and the complexities and “subtleties” in understanding the concepts of being at risk for a mental disorder rather than being diagnosed with one. Clinicians also suggested that clients might focus more on the clinician-client “fit” rather than on retaining information.

“*And they are sussing you out and getting a feeling for whether I want to talk to this person, getting a sense of the process, the atmosphere, rather than the content. You could be talking about anything, it's just a matter of ‘What's the rapport like? What's engagement like?'”*

To facilitate this engagement process, clinicians felt it was important to be “on the same page” as clients in terms of what clients wanted help with and what the service could offer. Addressing the concept of risk within this context was seen as something that could impair engagement and that timing was an important consideration to minimize this. There was general agreement, however, that providing information about risk was ethically correct.

Psychiatrist participants drew analogies between PACE clients and risk assessments for physical health conditions, for example saying that they would not consider withholding information about the reasons for, and potential outcomes resulting from a pap smear. There was a concern that withholding information was therefore potentially stigmatizing. Psychologists agreed that in order to make an informed decision, information about risk must be provided. However, they also felt that the rapport between client and clinician was more important than in physical health, and often part of treatment itself in mental health settings. Therefore, addressing risks and discussions about the potential for developing psychotic disorders must be done in considered ways. Clinicians didn't always feel like they achieved this balancing act, and after discussion about each of these issues, one clinical psychologist reported how this tension played out in his own work:

“*Well, there's a tension and it's an obvious one, to say that the psychoeducation or discussion about psychosis and transition, when we deliver that and how we deliver it, and sometimes it feels a bit inappropriately like playing God if we think we can only give it in this way to these people at this time. Whereas, if it was for a physical illness, we would generally have… very little reservation about giving the most comprehensive information… for some reason… there can be a kind of squeamishness, in case we cause some stigma or trauma (or) we increase the vulnerability by the discussion somehow, or by talking about something by naming it, we may make it happen or make it more likely to happen, which I guess rationally that's ludicrous. (But) I can act in that way I think. I can enact that in my practice at times. So I do think that for some people, there is a… timing issue, but sometimes we can put kid gloves on a little bit about transition, when perhaps we shouldn't. We should think about it in the same measured way we think of all psychoeducation.”*

Language was one important related consideration, and some clinicians described their practice of avoiding the term “ultra high risk.”

“*I think one of the questions with the language, like, while I talk to people about potential risk and so forth, I never use the term ‘ultra high risk.' For me, that is something that's read in journals and professions kind of communicate around but it is not the language that I would use in the room… ‘ultra high risk' sounds so imminent.”*

Alternatives to this phrase included “risk,” “chance,” and “symptoms worsening.” At the same time, although clinicians wanted to acknowledge that they were trying to prevent symptoms from worsening, they were also careful not to frame the event of transitioning to psychosis as “terrible.” One participant compared discussion about transition to that of relapse prevention in first episode psychosis, where clinicians aim to “balance that message with the client, so that relapse, when it comes, is not catastrophic.”

#### Assessment Phase, Including Establishing Priorities for Treatment

When discussing the assessment and treatment decision making phases, clinicians raised a number of factors that made it necessary for them to take a flexible and individualized approach with each client. Firstly, although clients all met the UHR criteria in order to be referred to the PACE Clinic, there was great diversity reported in both the different groups in the criteria (i.e., vulnerability group, attenuated psychotic symptoms groups, and the BLIPS group) and the individuals meeting criteria for each group or combinations of groups. This included differences in experiences and reasons for referral. Most notable was the differentiation between whether or not a client was experiencing attenuated positive psychotic symptoms.

“*Yeah, it's kind of like the UHR stuff is ‘why you are here' but what we do when you are here, it can be anything. we don't always focus on attenuated (positive psychotic) symptoms or sometimes there aren't any attenuated symptoms. When people come in on a family history, you don't work on psychotic symptoms. So the focus of care is often not attenuated symptoms.”*

The different ways in which clients accounted for their experience also contributed to this need to personalize care, and sometimes created challenges for clinicians when trying to work in a client-centered way.

“*Each and every person comes with a different explanatory model of their… symptoms, the cause and their prognosis and the treatment options. So sometimes I find it very difficult to incorporate their model and our model and make a common model for the treatment…”*

Language was seen as an important consideration when reconciling different understandings or “frameworks” for understanding the experiences of clients. “…trying to get a shared language” was valued in this phase of assessment and treatment decision making because, as one clinician noted, “you can't even negotiate a decision if you are not speaking the same language.” Some clinicians were willing to relinquish their own way of describing mental health issues.

“*I try and sometimes distance myself from the (professional) language, so that I can open up the idea that I am very happy to have their understanding… it's really important for us to have a shared way of talking about things.”*

Another issue that shaped the nature of assessment and treatment decision making was the fact that treatment didn't focus on one thing, which meant that a structured approach was not well-regarded by clinicians. However, one caveat to this flexible and personalized approach was that there was a possible disadvantage in focusing solely on the presenting problem of each client and not addressing the risk of psychosis. In doing so, clinicians were concerned that clients would not be able to make a fully informed decision about treatment: “In order for it to be informed decision making, you need to have information (about risk).” It was also noted that although the treatment might focus on other symptoms or life stressors, that this in itself can reduce the risk of psychosis and should be framed in such a way.

“*These symptoms might have a complex and subtle relationship with each other. So although we are not working directly on attenuated (psychotic symptoms), they might improve anyway as we gain, say, on depression or something like that. That is a message that can be communicated too.”*

Overall, addressing risk was seen as necessary but inherently complex.

#### Initial and Ongoing Treatment Decision Making

When it came to making decisions about treatment, aside from the heterogeneous nature of the individuals meeting the UHR criteria, additional issues included any past experiences of symptoms and treatment clients had. This included either directly (i.e., personal experiences) or indirectly (e.g., observing the experiences of a family member). This was seen as most notable in relation to medication,^1^ where both direct and indirect past experiences as well as expectations were seen to play a role.

“*And there is a very perhaps undue influence or undue emphasis on what the medication should be able to achieve in a short period of time. If it is not (working), then it's ‘junk' and ‘you're a fraud.' So it's difficult sometimes.”*

In response to this, clinicians were generally supportive of the idea that clients should be routinely informed about “stats around effectiveness, efficacy, but also how long they are likely to be on it.” This was seen as particularly important when multiple clinicians are involved in the care of clients and where information provision needs to be consistent. However, clinicians were also aware of both inadvertent and strategic “underselling” of the length of time clients may need to take medication for. One message that was seen as important, however, was the limitations of the emerging evidence base for the area.

“*I think the other thing, in particular (with) UHR and psychosis, (is) there's still a hell of a lot that we don't know. So whatever education we give, we have to include that… I think in this particular area, that we need to be quite clear about… where we are (at) with our evidence base.”*

In line with the provision of evidence-based information, clinicians also reported favoring a collaborative approach to making decisions with clients. They saw it as essential for clients to be “on board,” and felt that this was necessary for treatment to work.

“*It is also tricky, isn't it? Because treatment is only going to work if you have got the young person on board. You see the young person for an hour in that week (but) treatment needs to occur outside of that 1 h, otherwise it's not going to be effective. At the end of the day if you don't have the young person on board… you can't do anything. You think about graded exposure, you think about [Cognitive Behavioral Therapy], you think about [Cognitive Analytical Therapy]—all these therapies rely on young people—and medication—rely on them doing things outside that 1 h.”*

Although limitations to achieving this were noted, including the basic structure of treatment at the PACE Clinic (i.e., in general, weekly appointments with a case manager and medical reviews as necessary) and the ability of clients to participate in decision-making processes.

“*I strongly encourage it to be a collaborative experience and try to lay out lots of the options at the start (saying) ‘the best way I can help you is if I can see you weekly because then I can get a good sense of things, I can monitor things and we can maximize the time that you have got available (in the service)' but for some people it's very difficult for them to make a decision and understand what the implications of the decision are.”*

### Clients' Experiences and Beliefs About Treatment Decision Making

**Figure 2:**
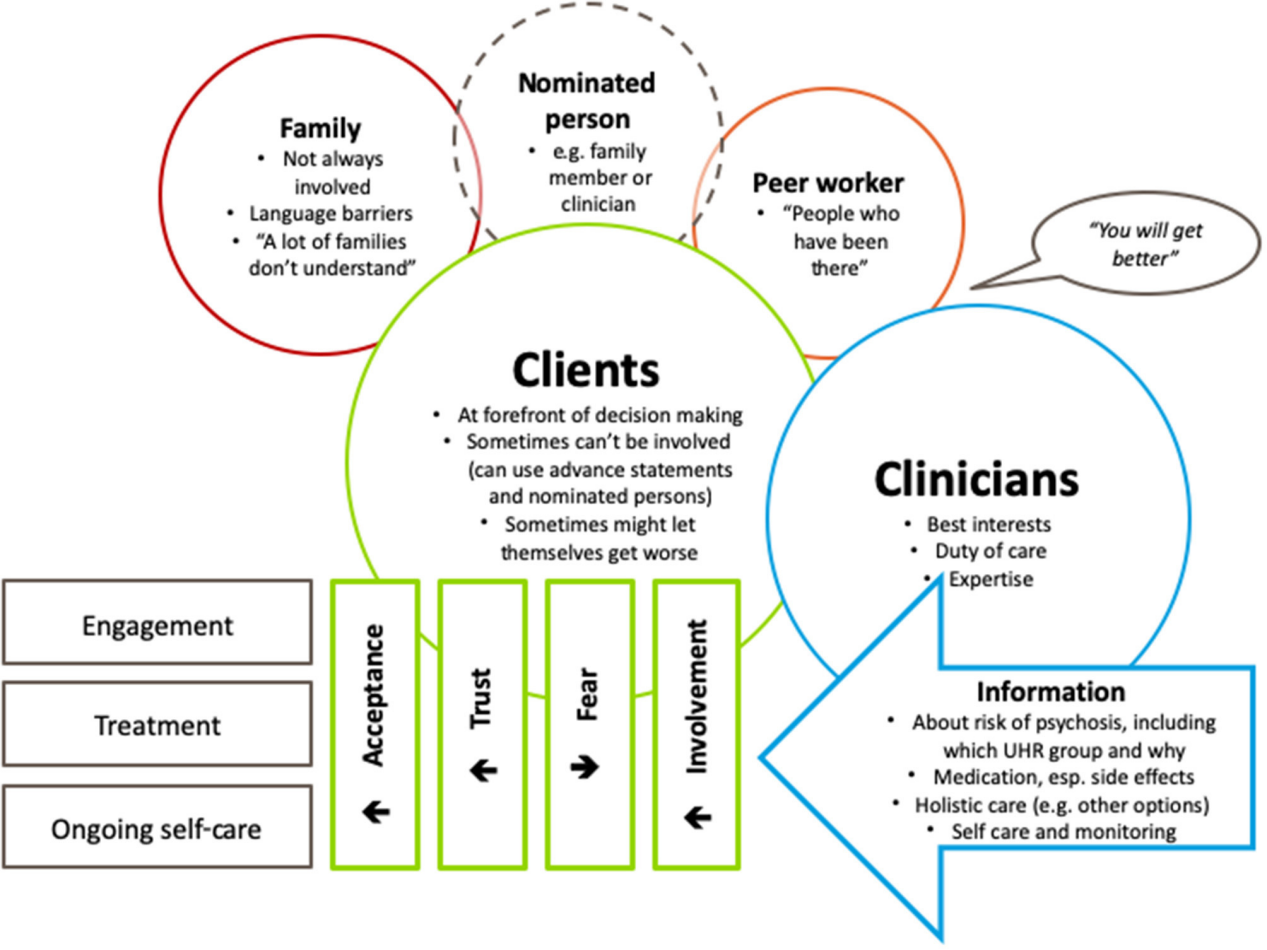
Thematic map of client reported experiences and beliefs about treatment decision making for young people who meet the “Ultra High Risk” (UHR) criteria.

In contrast to the clinician participants who focused on the decision-making processes in relation to a linear progression through assessment then initial and ongoing decisions, client participants more strongly referenced the context in which the decisions were made. This was particularly so in relation to feelings of initial resistance and then acceptance, be it acceptance of symptoms, of diagnoses (where relevant) or of different types of treatment. Throughout this journey, clients highlighted the need to be involved, for their clinicians to be involved and for clients themselves to be equipped with the knowledge and tools to take care of themselves (see Figure 2). Other people potentially involved in decision-making processes (family members, nominated persons, and peer workers) were seen as peripheral by most clients and are discussed below. In general, clients focused on their roles and the roles of the clinician.

#### Involvement in Decision Making

In line with the clinician data, clients acknowledged the importance of their contribution to the decision-making processes.

“*I think the client should sort of be at the forefront of the decision making, so when clinicians come to the client with the treatment plan, I think the patient should be consulted and their decision or their input should be taken very highly and should be understood as much as possible.”*

Client involvement was seen as important for engagement, particularly in relation to accepting the proposed treatment plan.

“*I think as well with the client, if they're more involved with (deciding about) their treatment I think they'll be more accepting of it as well. So, they won't be as hesitant as they are… when the clinician tells them exactly what to do. If they get consulted more they might be more willing to get better.”*

In terms of how to achieve this, “being told about alternative pathways” and being able to visualize the possible outcomes were suggested. Although this was seen as important, clients stressed that this needed to be in the context of a recovery framework in that it had to focus on the individual needs of the person and how they perceived “getting better.” Encouraging the client to accept their situation, including symptoms and treatment, was seen as a key part of this process that could be facilitated by involvement and the sharing of information, thereby building trust in the clinician.

“*I think it's important to let the person know that if they want to get better they can get better. That they can come in with their symptoms wanting to recover… you can tell them all the treatments, all the risks and benefits, but in the end if the person is not capable of accepting themselves and saying ‘okay yeah I can deal with this' it becomes confusing and harder. Saying ‘do you want to get better?' and from there onwards working on therapy that works for them and seeing what works for them.”*

#### Information Provision

Alongside the perceived responsibility of the client to work toward getting better, was the responsibility of the clinician to provide information about being at risk for psychosis, potential harms, and benefits of treatment options (including medication), psychological therapies (e.g., what type they were engaging in), alternative therapeutic options, hospitalization, and how clients could take care of themselves both outside of sessions during their time at the service, and also beyond their time at the service. In terms of information about risk, clients wanted to know why they were at the PACE Clinic, that they met one of the groups from the UHR criteria and *why* they met criteria. There were mixed experiences of what information was provided to clients about risk. For example, one client reported that they were told “immediately,” another said that they probably were told but could not remember, and a third was told that they were at the “right clinic” because they had a family history. Another client reported that they didn't know the name of the clinic, just where to go.

*Client 1: “There were a lot of words that we were never told”*

*Facilitator: “That's what I'm interested in”*

*Client 2: “Because (when) we came to Orygen we don't really get to know…”*

*Facilitator: “Did you know what clinic you were in?”*

*Client 1: “I just knew that I turned right at the corridor”*

*[Group laughter]*

For those clients who were told about their risk of developing psychosis, this information could be reassuring.

“*I have siblings that have psychosis. I have parents that have psychosis, and my mum has other mental health issues as well so, and I was also experiencing some delusions and symptoms of psychosis I guess. So, I understood why I was in the clinic. It was scary for me as I… I didn't want to be in there. I didn't want to have psychosis. But after understanding and knowing what was happening, and where I was in the service, I did feel better about it. Now, it's not that scary.”*

Clients showed a desire to have information provided to them unfiltered. This was particularly the case in relation to medication. Clients didn't want clinicians to “beat around the bush,” which they felt would lead them to “find out down the track the hard way.” Clients also resented experiences where they perceived clinicians to be withholding information, which affected their ability to trust them.

“*And sometimes I find that, you know, the psychologist, or the psychiatrist, sometimes I question them. Are they lying to me? Because everybody, my relatives, my friends who haven't seen me for a couple of months will be like, ‘how much weight did you stack on?' and I'm thinking my doctor never told me that. Are they lying to me? […] then I start resenting my doctors, and thinking are you lying to me because you only want me to keep taking these pills?”*

Most clients had experienced side effects (e.g., feeling “slummed out,” restlessness), with the most commonly reported side effect being weight gain. Several clients reported information about the risk of weight gain being withheld from them to encourage adherence. There were variations in the degree to which clients felt this was justified. Although most clients felt that the benefits of the medication outweighed the risks (e.g., “I'd rather have side effects than be how I was before”), there was a general desire for more information to be provided from the outset, and a strong desire for a holistic approach that included being told about alternative treatments and ways to live with both side effects and ongoing symptoms.

Clients also wanted information to be ongoing, interactive and meaningful. They valued instances when clinicians had facilitated self-monitoring, as “you see (treatment's) working or whatever you're doing is working, the medication's working, everything's working.” This tied in with client's perceived need to be able to take care of themselves outside of sessions and beyond their time at the service. This was achieved in a number of ways, for example with subjective scores.

“*We did have goals every 6 months, we would check on those goals and see how they were progressing. I think a good thing we did was um they gave me like a scale, I don't know if you guys did this as well, but they would just they would ask me how I was feeling on a scale from one to ten, compared to 6 months ago… I thought that was really helpful and a big thing they did was focus on things that we could achieve.”*

#### The Role of Clinicians, Family Members, and Peer Workers in Decision Making

In terms of knowing how to be involved in making decisions, clients described clinician practices that they felt involved them (e.g., tailored goal setting), and formal mechanisms provided by the service (e.g., the use of advance statements and nominated persons forms). However, clients had experienced both benefits and challenges of such approaches. Where there was trust, the use of nominated persons was perceived to have the potential to facilitate person-centered care:

“*I've nominated at the moment. I'm planning to have my old psychologist from Orygen to be my nominated person because I have full trust in him being off the relationship that we built in the past and he was upfront into medication and stuff. He sat me down and walked me through medication and decided all that kind of thing. So, to have him as a nominated person and to trust him in terms of what my treatment should be I think that really will definitely help with my treatment in (the) future and puts me at the forefront of my decisions and a psychologist created plan.”*

However, barriers existed for others, including a lack of people to nominate, and the suitability of these mechanisms for early intervention settings.

“*The problem with the advanced statement and the nomination thing is that with Orygen Youth Health, a lot of us are just, we just, that's the first time that we have become unwell. It's hard to choose a nominee sometimes, or to have an existing nominee and to have an advanced statement.”*

The challenges related to the use of advance statements and nominated persons processes were not the only caveats to the importance of involving clients in making decisions about their own treatment. Clients noted that there were times when they were unable to be involved (e.g., when experiencing psychotic symptoms) and that if decisions were left entirely to them then they would sometimes choose to let themselves get “worse,” which they wanted to be taken into consideration. In relation to this, clients were keen to stress the importance of the clinician's role in making decisions about treatment.

“*Well, at the end of the day, the clinician is the person who treats people all the time, has the qualifications, they know the most about the subject. I mean while, while the person's own personal problems should come into it obviously because you know, everyone's mind is different, I think at the same time, trying to, if people have too much if a person has too much input in treatment they could, actually make themselves worse as opposed to better.”*

Although clients felt that it was useful to have clinicians taking an active role in making decisions together with clients, the role that family should play was more contingent on certain factors. One client did not have any family who lived locally, and another said that it was difficult for her parent to be involved due to language barriers. There was also a concern that not all families were understanding and supportive.

“*I don't think it should be left up to the family, because my family, lots of families don't understand it. They don't see it, they think becoming unwell is being physically ill.”*

At times of crisis, some clients found a coordinated effort between families and clinicians useful for keeping them safe and making decisions they felt unable to participate in (e.g., hospital admissions).

Regardless of who was involved in the decision-making processes, clients wanted the opportunity to feel hopeful about the prospects of recovery. They described an interplay between information provision, framing of information, and fluctuating stages of engagement with the service and treatment. Ultimately, they felt a tailored approach according to the needs and experiences of the client was necessary to promote meaningful engagement and recovery.

Clients wanted to have something more than just being told that they could get better—they wanted to believe it too, and they suggested that visualizations (e.g., mapping out possible trajectories) and meeting with peer workers as some examples that could bring to life the possibility of recovery.

“*I think that people should be told they can get better. I mean it was told to me but I didn't believe it. I guess that's why we have peer support workers and people who have been there but yeah someone telling them that they can get better because it does feel like you're going to die and your life's over.”*

### Phase Two: Pilot Testing of the Decision Aid

In total, *n* = 10 client participants and *n* = 6 clinician participants were involved in the piloting of the decision aid; however, client-rated data are only available for *n* = 9 client participants as one participant chose not to complete the questionnaires but gave permission for their clinician to provide the clinician-rated data. Clients were aged between 16 and 23 years (mean = 19.7; SD = 2.3) and 6 (60.0%) were female and 4 (40.0%) were male. Clinicians were aged between 30 and 42 years (mean = 34.5; SD = 4.2) and were all female. Clinicians had been working in their respective disciplines for between 4 and 10 years (mean = 6.3; SD = 2.5). Five of the clinicians were clinical psychologists and one was an occupational therapist; three were in senior roles.

#### Decision Related Outcomes

Scores on the SWD scale ranged from 12 to 29, with a mean score of 23.1 (SD = 5.3), indicating variability within the sample, but on average relatively high levels of satisfaction. These scores include participants who were unable to decide and one participant who was an outlier, as their scores indicated they had high decisional conflict and were not satisfied with the decision. This same participant provided minimal responses to the open-ended feedback questions (e.g., “no” and “boring”). Overall, all but two client participants reported being able to decide on a treatment option; one client participant reported still feeling undecided and another said they were unsure if they were decided. All treatment choices involved either CBT, supportive therapy or both, plus eight of the nine clients chose to treat their mental health challenges in general and two clients chose to take Omega 3 tablets. Both client and clinician participants unanimously reported that the treatment choice was in line with the client's preferences. At follow-up (approximately 6 weeks post decision), there were clinician-rated data for seven client participants. All but one participant continued with their treatment as intended; one client experienced first episode psychosis and their care changed accordingly. All but one client had failed to attend sessions, which is not unusual for the service. Several additional treatments and related options were noted, including inpatient admission, different psychological therapies (e.g., schema therapy, cognitive analytical therapy), medications, peer support, alcohol and other drug counseling, and “systems work” (liaising with the school, family, and social services).

#### Feedback on the Decision Aid

Client and clinician participants were invited to provide feedback on each section of the decision aid, including how useful they found it, whether the information was relevant to them; the appearance; and what they would change about the section. Results are summarized in Table 1. Clients and clinicians were generally positive about each section of the decision aid. One exception to this was clinician feedback on the “Treatment Options” page, which raised some concerns about its content and practical use. Clinician participants highlighted a mismatch between the options presented and what was offered at the service.

**Table 1:** Feedback from client and clinician participants on each section of the decision aid.

	**“Information, factsheets, and resources” page**	**“What matters to me” page**	**“Am I at risk?” page**	**“Treatment options” page**
**Client Feedback**				
Usefulness	• 1 neutral response • 1 negative response (not useful) • 7 positive responses, including ease of navigation, amount and type of information, practical application e.g., “It is a useful starting point for a conversation” e.g., “It lets the people know how I want to be treated”	• 1 did not use • 2 didn't find it useful e.g., “made me feel like getting over these things was really easy, when in reality it's the hardest thing I've ever done” • 6 found it useful e.g., “You can express what you like and don't like”	• 1 negative • 8 positive e.g., “was quite detailed in what an at risk mental state is” e.g., “because it's a question I ask myself”	• 2 didn't find it useful e.g., “left with doubts” • 1 appreciated finding out about treatment options they weren't aware of (e.g., Omega-3 fatty acids)
Relevance	• All positive responses, including noting that the diagrams and facts were useful, as were the personal stories e.g., “It was good hearing people's personal experiences and seeing stats”	• 1 negative: “I didn't agree with some things and I found that most of it was already what I thought” • 8 positive, including that the content related to their personal circumstances e.g., “I liked being able to find out what is important to me”	• All positive responses, including that it was reassuring, told them “what I need to know about what could potentially happen,” and they found it useful to read about “people with my condition”	• All clients found it relevant e.g., “I will be going through these treatments at some point and it explains what they are,” e.g., “it really helped me understand there's more help than just counseling.” • One client found it reassuring to be provided with information, saying “I have been terrified of institutions since opening up about my condition. It's nice to know what's actually going on.”
Suggestions	• More interviews • Larger font size • Additional links to resources • Additional interactive components	• Boring • Clearly displayed • Easy to navigate • More options and categories	• Additional information • More interactive features	• Additional information e.g., “personal experiences of success stories for each treatment” • More information about side effects of medication • More interactive components • More color: “a tad bland”
**Clinician Feedback**				
Usefulness	• All positive, including that it looked good, was engaging, easy to navigate e.g., “range of topics and different mediums useful” e.g., “lots of info in one place, easy access, videos were good option”	• All positive apart from one who replied “somewhat” but did not elaborate; two could not remember section e.g., “very engaging” e.g., “Like videos and consumer testimony”	• All positive but one clinician noted they were unsure the client understood the risk concept e.g., “Useful to have visual ways of presenting this concept” e.g., “Diagram was good [at] showing increased risk without being scary”	• 2 positive e.g., “Yes, able to talk about different options” • 6 conditional responses (e.g., “somewhat,” “didn't find it as useful”) and related caveats about mismatch between options presented in the decision aid and what the service can offer e.g., “need to be careful that it doesn't set expectations in [service] there is a standard package of care eg don't offer omega”
Relevance	• All positive apart from one participant who said “mostly” but didn't elaborate e.g., “helpful when providing psychoed to young person” e.g., “yes emphasized drop in functioning yet hopeful about recovery”	• All positive except one negative “didn't seem as relevant as first section” and one who could not remember the section e.g., “[very] helpful to explain stress-vulnerability model in a fun way” e.g., “animations and visual interactions are most useful”	• All positive, including “ARMS focus good” and “relevant to PACE cohort, visuals helpful to explain concepts” • Three caveats, including that “less text is more engaging,” that it was a “difficult concept to convey and needed more explanation,” and that “young people still find the ‘longitudinal' paradigm less of ‘relevance' than the here and now”	• 2 positive e.g., “Yes, given I had discussed some of these options already with the young person” • 6 noted caveats around including Omega-3 fatty acids as a treatment option when it wasn't available at the service • 1 noted that more recent evidence (not incorporated into the clinical practice guidelines) was not reflected in the decision aid • 1 noted the limitations of the decision aid for young people with other mental health challenges e.g., “Mostly, I'm unsure how to talk about use of fish oil and unclear what current recommendations should be. Also one client had BPD too so also talked about CAT” e.g., “We don't usually present fish oil as an option and the diagram presents it as very effective whereas neurapro didn't show this so it feels very prominent in the choices given, and at the point that the decision aid was used it's a little hard to describe therapy as a choice between supportive and CBT and to differentiate that from treating mental health in general as often these are combined”
Suggestions	• 4 said no changes required • Additional information required for some clients • Too much information to cover in one session • Balance provision of information with engagement • Include fact sheet on role of general practitioner • Preference for videos over “boring” fact sheets	• Make it less simplistic for some clients • Being able to print out section • Interactive version for client to use at home or handout to accompany online decision aid used in session	• Try and emphasize how here and now affects the “at risk” concept • Include audio to explain each graph • Risk communication graphs were too big—suggestion to reduce them to be out of 10 people instead of 100	• Additional details about different therapies • Have a function so that clinicians can tailor it to the young person and/or what is available at the service • Additional details about options beyond the clinical practice guidelines (e.g., psychosocial recovery groups, vocational support, medication, family work)

## Discussion

This project sought to develop an online decision aid for young people at increased risk of developing a psychotic disorder. To date, the majority of decision support tools for young people have been designed for parents to make decisions for their children (47); this study focused on the young people (adolescents and young adults) as the decision makers themselves. This project contributes to a growing number of studies that demonstrate young people in this age group can be supported as the primary decision makers (35, 37, 48). This decision aid was developed in the context of significant academic and clinical debate about the ethical merit of identifying young people who meet the criteria for being at increased risk for developing a psychotic disorder, informing them of this risk, and delivering interventions to delay, prevent, or otherwise ameliorate the impact of first episode and/or recurrent psychotic episodes [e.g., (22, 23)]. We have previously proposed SDM as a practical way in which to address these ethical issues (19), and the current study sought to provide decision support to clients to allow them to be active participants in deciding whether or not to access treatment, and if so, collaboratively choose their preferred treatment.

This project was conducted across two phases: phase one involved qualitative focus groups with past clients and current clinicians at an early intervention service; and phase two pilot tested a decision aid at an early intervention service. Phase one highlighted the similarities and differences between the perspectives and frames of reference that clients and clinicians had about treatment decision making in this area. For clinicians, this was focused on entry to the service, time with the service, and discharge from the service, whereas for clients these decisions were described more in relation to the context of the time at the service in their overall lives. Phase two highlighted that although the decision aid was well-received overall, it had some limitations in terms of utility and relevance.

A consistent theme for client participants across both phases was the desire for the possibility of recovery to be brought to life through peer support (phase one) and personal stories (phase two). This highlights the value that young people placed on lived experience and the contribution to both decision-making processes and treatment itself. Although this pilot trial was focused on decision making as a collaboration between clinicians and clients, it is possible for youth peer workers to be involved in supporting young people to make decisions about mental health care (49). Exploring this model for young people at increased risk of developing psychosis may not only reduce decisional conflict, but also enhance the degree to which young people feel involved in decision making with clinical staff (35).

Pilot testing of the decision aid showed mixed results for which sections client and clinician participants found useful and relevant. A number of clinicians felt that certain sections of the decision aid (e.g., treatment options) were limited in their utility and relevance given that one treatment option (Omega 3) was perceived to be unavailable at the service. We note that some clients were listed as having chosen this treatment option, but reasons for this discrepancy were unclear. It is possible that clinicians were aware of the specific composition used in clinical trials [e.g., (50)] and how that was not available at pharmacies at the time, whereas others considered readily available fish oil tablets to be sufficient to recommend to clients. There was also concern raised that more recent, at the time unpublished, results of a trial that involved the PACE clinic failed to replicate the effectiveness of that treatment option (51).

When young people present to services for psychosis prevention, their priority for treatment may not be reduction of attenuated psychotic symptoms (52, 53). Although interventions for this cohort have been shown to reduce psychotic symptoms and rates of developing a psychotic disorder, they have not been effective in other important outcomes such as symptoms of depression or functioning (13). The findings of phase two highlight the limitation of support tools for specific decisions, in that they don't account for the specific decision in the context of all of the related decisions the user is facing. Comments made by clinicians on the usefulness and relevance of the decision aid highlighted how complex the overall treatment decisions were for many clients. The types of treatment options that client participants ended up receiving included options beyond the scope of the decision aid (e.g., referral to alcohol and other drug services, specialized psychological therapy for personality disorders). Although they could be collectively labeled as “treatment for other mental health issues,” which is a recommendation of the clinical practice guidelines and therefore an option presented in the decision aid, it was not possible to include all possible referral options for related decisions. This highlights the need to consider how a decision aid for a specific decision (or in this case, two decisions) might be embedded in an overall collaborative approach to decision making. Clinical trials often test interventions for narrowly defined conditions, resulting in a lack of evidence about what works for comorbid presentations, affecting translational resources such as clinical practice guidelines and decision aids (54). In mental health, and especially in youth mental health, there is a lack of data to inform decisions for people experiencing any combination of emerging or established mental disorders, personality disorders, and/or substance use disorders. This limits the degree to which complex decisions can be supported by these types of decisions aids, and emphasizes the importance of general decision support interventions, such as generic decision aids (to support any decision) [e.g., (35)], training in SDM for clinicians with well-defined core competencies (55), and interventions designed to increase mental health literacy, empowerment, and decision making skills for clients and their families [e.g., (56)].

Other approaches involving clients were discussed by client participants in phase one, namely the use of advance statements and nominated person forms. These forms represent legal mechanisms designed to uphold the rights of people to make decisions about their own mental health care (57). However, participants felt that the utility of these were limited in early intervention services where young people may experience being acutely unwell for the first time, so aren't able to express their preferences in advance or make informed experience-based choices. A qualitative study of clients, caregivers and clinicians from the first episode psychosis service at OSPs (EPPIC) showed that these tools were not commonly used in the clinic, and a number of barriers to use were described by all three participants groups (58). Nevertheless, participants were equally enthusiastic about the potential of these tools and about collaborative decision-making approaches in general.

These findings have direct and indirect implications for early psychosis services. Ensuring that young people have positive experiences with mental health services requires strong, positive relationships with clinicians that are genuinely collaborative and prioritize the needs and wants of clients (59). This is important not just at entry to the service, but is critical for meaningful engagement across the duration of care (60). Collaborative approaches, such as SDM, are likely to enhance the strength and quality of relationships, but need to be embedded in the overall culture and policies and practices of youth mental health services; one approach alone will be insufficient (61).

A strength of the current study is that it contributes to the neglected area of how to involve young people in making decisions about their own mental health care. A limitation is that we did not include caregivers (e.g., family members) as participants. Future research should incorporate these perspectives, as there are likely to be unique contributions to understanding how decisions are made within and beyond clinical sessions. Another limitation is that this was a pilot trial and not designed to test the effectiveness of the decision aid. Given that across healthcare settings decision aids consistently demonstrate effectiveness in reducing decisional conflict (27), such an effectiveness trial may not be the most important research question to pursue. It may be more fruitful to focus on how decision aids can be part of a larger, more complex intervention designed to embed SDM across a service or service system. This will require the use of implementation science to fully understand the barriers and enablers to creating sustainable change. Finally, this study is limited by the small number of participants and the lack of the proper co-design methodologies we would now use and which may prevent and/or address some of the critical feedback from participants more rapidly.

Overall, this study highlights the importance of incorporating SDM practices into youth mental health settings when working with young people at UHR of psychosis. The decision aid that was piloted demonstrated utility within this population and while some limitations were highlighted by both clinicians and client participants, in general it was found to be both useful and relevant in supporting young people to make decisions about their treatment options.

## Data Availability Statement

The raw data supporting the conclusions of this article will be made available by the authors, without undue reservation.

## Ethics Statement

The studies involving human participants were reviewed and approved by Melbourne Health Human Research Ethics Committee. Written informed consent to participate in this study was provided by the participants' legal guardian/next of kin. Participants who were aged 18 years or older provided their own consent.

## Author Contributions

MS, AP, and BN contributed to the conception and design of the study. MS, AE, and AM recruited participants and collected data for the study. MS and AM performed the qualitative analysis. MS wrote the first draft of the manuscript. MB wrote sections of the manuscript. All authors contributed to manuscript revision, read, and approved the submitted version.

## Funding

MS was supported by a Melbourne Research Fellowship from The University of Melbourne. This study was funded by a philanthropic donation from MessageMedia. The funder was not involved in the study design, collection, analysis, interpretation of data, the writing of this article or the decision to submit it for publication.

## Conflict of Interest

The authors declare that the research was conducted in the absence of any commercial or financial relationships that could be construed as a potential conflict of interest.

## Publisher's Note

All claims expressed in this article are solely those of the authors and do not necessarily represent those of their affiliated organizations, or those of the publisher, the editors and the reviewers. Any product that may be evaluated in this article, or claim that may be made by its manufacturer, is not guaranteed or endorsed by the publisher.

## Footnotes

^1^Although antipsychotic medication is not prescribed outside of research studies at the PACE Clinic, some clients may be on other medications for non-psychotic mental disorders (e.g., antidepressants).
