# Supplementary material for: Shared Decision Making With Young People at Ultra High Risk of Psychotic Disorder
Source: Front Psychiatry. 2021 Sep 16;12:683775. doi: 10.3389/fpsyt.2021.683775 (PMC8481955; doi:10.3389/fpsyt.2021.683775)

Supplementary Materials 1: Example screen shot from decision aid

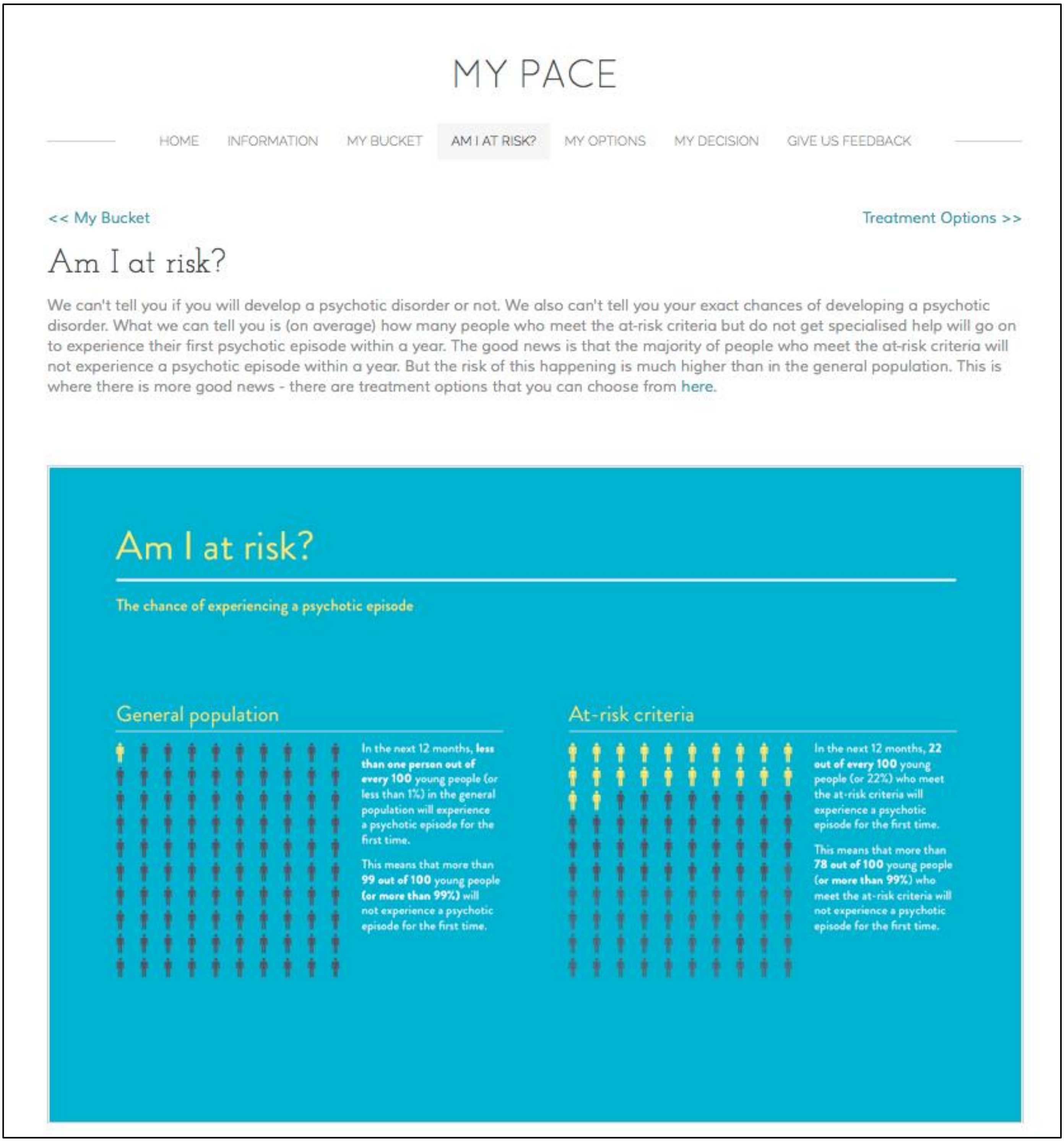

Supplement: Supplementary file 1 [file Image_1.pdf]
