# Supplementary figures and images for: Shared Decision Making With Young People at Ultra High Risk of Psychotic Disorder
Source: Front Psychiatry. 2021 Sep 16;12:683775. doi: 10.3389/fpsyt.2021.683775 (PMC8481955; doi:10.3389/fpsyt.2021.683775)

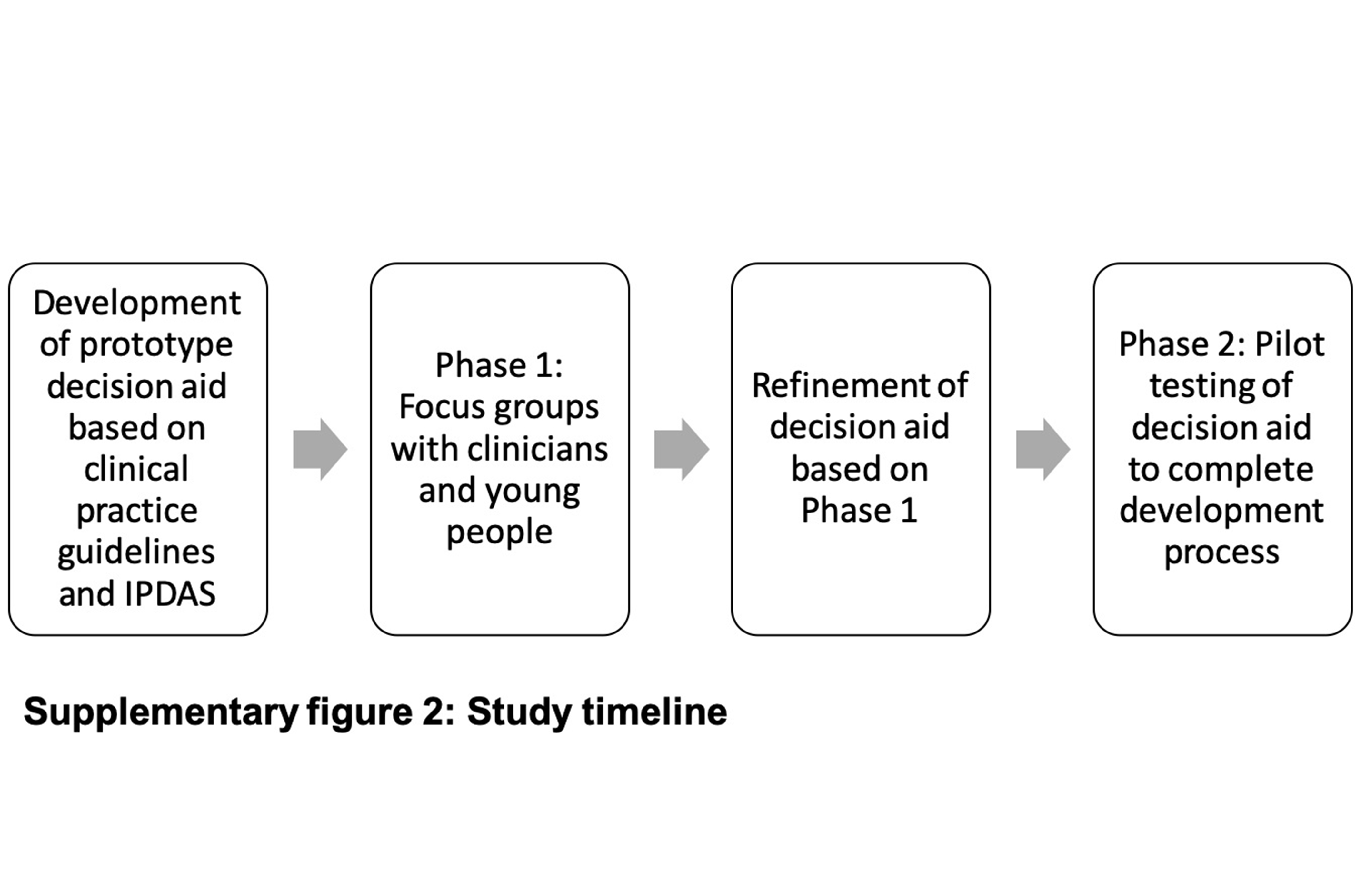

Supplement: Supplementary file 2 [file Image_2.jpg]
