# Supplementary material for: Shared Decision Making With Young People at Ultra High Risk of Psychotic Disorder
Source: Front Psychiatry. 2021 Sep 16;12:683775. doi: 10.3389/fpsyt.2021.683775 (PMC8481955; doi:10.3389/fpsyt.2021.683775)
Supplement: Supplementary file 3 [file Image_3.pdf]

Supplementary Materials 3: Example Focus Group probes

General discussion items

1. What are the key decisions faced by PACE Clients?
2. What are the relevant treatment options available to PACE Clients?
3. For each treatment option:
    a. What are the potential benefits of doing this option?
    b. What are the potential risks or harms of doing this option?
    c. What information do people need to know about this option?
    d. What can be difficult about making a decision about whether or not to do this option?
    e. What personal preferences and values (of clients) have you seen impact decision making for this option?

Decision aid specific discussion items

1. Home page
    a. What do you think of the *content* of this page? What would you definitely keep? What would you definitely change?
    b. What do you think of the *layout and design* of this page? Can you comment on whether or not it is user friendly? What other functions would be useful here?
2. Risk of transition page
    a. What do you think of the *content* of this page? What would you definitely keep? What would you definitely change?
    b. What do you think of the *layout and design* of this page? Can you comment on whether or not it is user friendly? What other functions would be useful here?
    c. Can you talk about the positive or negative aspects of the graphs?
3. Treatment options page
    a. What do you think of the *content* of this page? What would you definitely keep? What would you definitely change?
    b. What do you think of the *layout and design* of this page? Can you comment on whether or not it is user friendly? What other functions would be useful here?
    c. Can you talk about the positive or negative aspects of the graphs?
4. What matters to you page
    a. What do you think of the *content* of this page? What would you definitely keep? What would you definitely change?
    b. What do you think of the *layout and design* of this page? Can you comment on whether or not it is user friendly? What other functions would be useful here?
5. Information page
    a. What do you think of the *content* of this page? What would you definitely keep? What would you definitely change?
    b. What do you think of the *layout and design* of this page? Can you comment on whether or not it is user friendly? What other functions would be useful here?
